# IEg67 kDa Bovine Hydatid Cyst Antigen: A Candidate for Developing Sero-Diagnostic Assays for Cystic Echinococcosis, a Disease of One Health Importance

**DOI:** 10.3390/ani13050866

**Published:** 2023-02-27

**Authors:** Sakandar Khan, Jo Cable, Muhammad Younus, Muhammad Imran Rashid, Frank Hailer, Haroon Akbar

**Affiliations:** 1 Department of Parasitology, Faculty of Veterinary Sciences, University of Veterinary and Animals Sciences, Lahore 54000, Pakistan; 2 School of Biosciences, Cardiff University, Cardiff CF10 3AX, UK; 3 Department of Pathobiology, College of Veterinary and Animal Sciences, Narowal, Sub-Campus, University of Veterinary and Animals Sciences, Lahore 54000, Pakistan

**Keywords:** zoonoses, One Health, neglected tropical disease, *Echinococcus granulosus*, hydatid cyst, sero-diagnosis

## Abstract

**Simple Summary:**

Cystic echinococcosis is a world-wide zoonotic disease of food animals and humans. The disease negatively impacts food production, causes socio-economic hardship and is an animal welfare concern. Here, we identified and deployed a potential candidate antigen, iEg67 kDa crude antigen, for ELISA kit development that improved diagnostics for cystic echinococcosis in both cattle and buffalo, with 100% sensitivity and 93.8% specificity. The next steps are to validate and optimize this 67 kDa protein-based ELISA kit for sero-diagnosis of cystic echinococcosis in other species of food animals.

**Abstract:**

Cystic echinococcosis (hydatidosis) is a world-wide zoonotic disease of mainly humans, livestock and dogs, caused by *Echinococcus granulosus*. The disease can negatively impact food production and animal welfare and causes socio-economic hardship. Here, we aimed to identify the local bovine hydatid cyst fluid (BHCF) antigen for developing a sero-diagnostic assay to be used for the pre-slaughter screening of food animals. In total, 264 bovines approved for slaughter in Pakistan were subjected to serum collection and post-mortem screening for hydatid cysts. These cysts were assessed microscopically to assess fertility and viability, and by PCR for molecular confirmation of species. A BHCF antigen was identified from positive sera via SDS-PAGE, confirmed by Western blot, and quantified via a bicinchoninic acid (BCA) assay. The quantified crude BHCF antigen (iEg67 kDa) was then used in ELISA screening to test all sera collected from known positive and negative animals based on hydatid cyst presence/absence. Of the 264 bovines examined, 38 (14.4%) showed hydatid cysts during post-mortem examination. All of these individuals, plus an additional 14 (total: 52; 19.6%) tested positive based on less time-consuming ELISA examination. Based on ELISA, occurrence in females (18.8%) was significantly higher than in males (9.2%) and was higher in cattle (19.5%) compared to buffalo (9.5%). The infection rate increased with age in both host species: cumulatively, 3.6% in animals aged 2–3 years, 14.6% in 4–5-year-olds and 25.6% in 6–7-year-olds. The occurrence of cysts in cattle was significantly higher in the lungs (14.1%) compared to their livers (5.5%), whereas the opposite was true in buffalo (6.6% livers, 2.9% lungs). For both host species, most cysts in the lungs were fertile (65%), while the majority in the liver were sterile (71.4%). We conclude that the identified iEg67 kDa antigen is a strong candidate for the development of a sero-diagnostic screening assay for the pre-slaughter diagnosis of hydatidosis.

## 1. Introduction

Pakistan has a large proportion of farmers entirely dependent on livestock for their livelihood. Livestock has a major role in the economy of Pakistan, contributing >60% to agriculture, and showing an annual growth rate of 3.06% per year and 11.5% for gross domestic product (GDP) (Pakistan economic survey 2020–2021). The livestock are affected by a range of infectious diseases that adversely reduce growth rates and overall production, particularly affecting food animals. Cystic echinococcosis (hydatidosis) is one such zoonotic disease, caused by the cestode *Echinococcus granulosus* that infects both humans and animals [1] and has a cosmopolitan distribution [2]. The disease occurs in South America, Eastern Europe, Russia, East Africa, Central Asia, Iran, Iraq, Syria, Jordan, Saudi Arabia and Pakistan [3]. According to the Global Burden of Disease (GBD), 9186 symptomatic cystic echinococcosis patients were reported in Pakistan in 2016, and new cases reported in the same year totalled 2932 per 100,000 population (Global Burden of Disease Results Tool 2021). In many parts of the world, e.g., Mediterranean regions, the Americas, Asia, Australia, Africa, and Europe [4], echinococcosis is categorised as a neglected tropical disease [5].

Two types of hosts are responsible for the transmission of *Echinococcus* spp.: ruminants and canines. Transmission to intermediate hosts (sheep, cattle, buffalo and goats) occurs through the ingestion of cestode eggs expelled by the definitive host [6]. Dogs and other canines serve as definite hosts when they ingest offal contaminated with *Echinococcus granulosus* larvae [7]. Humans are accidental hosts for *E. granulosus*, acquiring the infection through close contact with dogs or by ingesting water or food contaminated with parasitic eggs [8]. As accidental or dead-end hosts, humans cannot transmit the infection. In the intermediate hosts, the parasite develops in a fluid filled hydatid cyst in soft organs, often the liver and lungs, and rarely in the brain, kidney and bone marrow, resulting in morbidity and mortality [9,10,11]. In humans, the infection causes bile duct obstruction and pleural fistula disorders [12]. To prevent and control cystic echinococcosis, methods applied to break the life cycle between the definitive and intermediate hosts include the deworming of dogs, meat inspection, not feeding infected offal to dogs and public awareness of the risks to humans [13]. Despite this, cystic echinococcosis is highly prevalent in ruminants within herd-keeping areas of the world [14], ranging from 53.9% in China [15] and 22% in Ethiopia [16] to 13.9% in Iran [17] and 12% in India [18]. The prevalence of echinococcosis in Pakistan ranges from 2.4 to 35% in different host species [1,19,20], and here the estimated economic loss caused by cystic echinococcosis is USD 1.65 per organ [21].

To control cystic echinococcosis, early diagnosis is essential [22]. The diagnosis of *Echinococcus* typically relies on post-mortem extraction of the parasites, followed by microscopy and PCR. Echinococcosis cannot be confirmed through clinical signs, and in many countries, there are limited or no microscopy and molecular facilities available for ante-mortem examination. Additionally, radiography and ultrasonography are ineffective in identifying newly formed cysts. Serological assays, on the other hand, could provide a powerful pre-slaughter diagnostic screening tool for cystic echinococcosis. This could reduce the spread of *E. granulosus* on farms and in the environment, important for the prevention of human infections.

Bovine and sheep hydatid cyst fluid (8, 16, 24 and 38 kDa) and goat hydatid cyst fluid antigens (60 and 245 kDa) have been previously studied for their potential usage in sero-diagnostic assays [23,24,25]. In Pakistan, hydatid cyst fluid antigens of goat, sheep, cattle and buffalo have been characterised [26], but so far have not been tested for use in sero-diagnostic assays. Hence, here we isolate the unique antigen/s from bovine hydatid cysts to assess their usefulness in developing an ELISA for the pre-slaughter sero-diagnosis of cystic echinococcosis in bovines.

## 2. Materials and Methods

### 2.1. Study Site and Selection of Animals for Sera and Cyst Collection

Sampling was conducted at a slaughterhouse in the district of Sheikhupura (Pakistan) in December 2021, where 960 bovines (cattle and buffalo, mostly dairy animals) were slaughtered during that month. Animals with the lowest body condition score were targeted in this study. A total of 264 cattle and buffalo individuals were tagged for collection of sera and for post-slaughter examination for hydatid cysts. Blood samples were collected from the jugular vein using sterilised 10 mL syringes (22G needle; Shifa) and stored in serum vacutainers. The tubes were transported on ice to the Department of Parasitology, University of Veterinary and Animal Sciences, Lahore, Pakistan, for further processing the next day. The serum was separated by centrifuging the samples at 3000× *g* for 20 min. The supernatant (serum) was collected and stored at −20 °C until further use.

### 2.2. Hydatid Cyst Collection

Following the slaughter of tagged animals, the carcasses were examined for hydatid cysts in the liver, lungs and kidneys. Cysts removed from the liver or lungs were placed individually in sterilised containers for transport to the laboratory and subsequent microscopic examination. No cysts were detected in the kidneys. The meat was not tested for the presence of *Echinococcus granulosus*.

### 2.3. Microscopy of Hydatid Cysts

Cysts with viable protoscoleces were classified as fertile and those with no protoscoleces were classified as sterile. To check fertility, fluid from each hydatid cyst was gently aspirated through a 1 mL sterilised syringe, and 0.2 mL was dropped onto a microscopic slide with a cover slip [27]. To assess the viability of protoscoleces, the fluid (0.1 mL) from each hydatid cyst was mixed with 0.1% eosin stain for microscopic examination at 400×, with only viable protoscoleces taking up the stain [28]. All slides were examined with an Olympus CX21FS1 microscope at 400× magnification.

### 2.4. Molecular Identification of Echinococcus Granulosus

DNA was extracted from the germinal layer and fluid of the hydatid cyst using the WizPrep™ gDNA Tissue Kit (Wizbiosolutions, South Korea, W71060-300). *Echinococcus granulosus* mtDNA ND1 primers were used: Eg1F81 5′ GTT TTT GGC TGC CGC CAGAAC 3′ and Eg1R83 5′ AAT TAA TGG AAA TAA TAACAA ACT TAA TCA ACA AT3’, which generate a 226 bp amplicon [29]. The PCR was performed in a T100 Thermal Cycler (Bio-Rad, Hercules, CA, USA) as described previously [30]. Briefly, a total reaction volume of 20 μL included 2 μL of genomic cyst DNA (11.5 ng/μL), 10 μL master mix 2X AmpMaster™ Taq (GeneAll^®^, Exgene™, catalogue number 541-001), 6 µL of Ultrapure™ DEPC water (Invitrogen, 750023) and 1 μL of each forward and reverse primer solution (50 μM). PCR conditions were as follows: initial denaturation step at 94 °C for 3 min, followed by 28 cycles of denaturation at 94 °C for 30 s, annealing at 59.8 °C for 30 s and extension for 1 min at 72 °C, and a final extension step for 5 min at 72 °C. PCR products underwent electrophoresis in 2% agarose gels (1 h at 120 V, current 400 mA) using an SYBR safe DNA gel stain (catalogue no. 2291850; Invitrogen, Waltham, MA, USA alongside 7 μL of DNA ladder (100 bp DNA ladder Gene-direx, Catalogue # DM001-R500), and were observed under a GelDoc 100 imaging system (BioRad, Hercules, CA, USA).

### 2.5. Preparation of Bovine Hydatid Cyst Fluid (BHCF) Antigen

Fluid was aseptically aspirated using a sterilised syringe (Shifa) from a hydatid cyst present in the liver of a buffalo, transferred to a 50 mL falcon tube, according to [23], and centrifuged at 7000× *g* for 30 min. Acetate buffer (0.005 M; pH 5) was used to dialyse the supernatant, and then the falcon tube was incubated overnight at 4 °C before centrifuging at 20,000× *g* for 30 min at 4 °C (ultra-high-speed centrifuge, Sigma 3K30). The precipitates were dissolved in 10 mL of phosphate buffer (0.2 M, pH 8) saturated with 40% ammonium sulphate and were centrifuged at 3000× *g* for 25 min. The supernatant was then incubated for 10 min at 60 °C and centrifuged again at 20,000× *g* for 1 h. The supernatant containing the soluble antigens of HCF was filtered with a 0.2 µm filter and stored at −20 °C [31,32].

### 2.6. SDS-PAGE and Western Blotting

The native antigens separated from the bovine hydatid cyst fluid (BHCF) were analysed via SDS-PAGE and Western blotting according to [33]. Briefly, each lane of a 12% *w*/*v* polyacrylamide gel was loaded with 30 μL native antigen (11.65 μ g/μL), protein-loading (Laemmli) buffer and protein marker (PageRuler^Tm^ Cat# 26614, thermo scientific), separated by 12% *w*/*v* polyacrylamide gel and transferred to the nitrocellulose membrane (NCM, 0.22 µm, Trans-Blot^®^ Turbo™ Midi nitrocellulose transfer packs # 1704159, Bio-Rad, USA) using the Trans-Blot^®^ Turbo™ transfer system (Bio-Rad, USA). The blots were cut into strips, labelled and blocked with (5% *w*/*v*) skimmed milk in tris buffer saline (20 mM Tris-HCL, pH 7.2, 150 mM NaCl). The positive serum collected from infected tagged animals (three buffalo, each with 2–3 hydatid cysts in the liver, confirmed after post-mortem) was used as the primary antibody. The strips were charged with the anti-bovine antibody (Anti-bovine IgG-alkaline phosphatase conjugate Sigma Cat # A0705-25ML) as the secondary antibody (1:20,000) after three washings. A chromogenic substrate (NBT/BCIP bio-WORLD, Dublin, OH, USA) was used as a colour marker.

### 2.7. Quantification of Native Antigen

The bovine hydatid cyst fluid protein was quantified using bicinchoninic acid (BCA) assay according to the manufacturer’s instructions (Cat. 786-570, G-Biosciences, St. Louis, MO, USA). OD values were recorded at 650 nm using a multiskan sky microplate (1530-800580) spectrophotometer.

### 2.8. ELISA

ELISA was performed according to [34] with minor modifications. Hydatid cyst fluid antigen (100 µL/well in 50 mM Na_2_CO_3_ coating buffer at the concentration of 5 μg/mL) was coated on to a 96-well flat-bottom polystyrene plate (JET BioFil, Hong Kong, China, Code # TCP011096) and incubated at 37 °C overnight. The plate was washed 5 times with 250 μL/well 0.001 M PBS/0.05% Tween-20, then blocked with 4% BSA in 0.01 M PBS (200 μL/well), incubated for 2 h at 37 °C and washed 5 times with washing buffers. The primary antibody (serum) was added to the plate at a dilution of 1:99. Two wells were coated with positive sera (confirmed after slaughtering and PCR) and two wells were coated with negative sera. The plate was incubated at 37 °C for 2 h and re-washed 5 times. The plate was then coated with anti-bovine antibody 100 μL/well (Anti-bovine IgG-alkaline phosphatase conjugate Sigma Cat # A0705-25ML) as the secondary antibody (1:5000), incubated for 2 h at 37 °C and then re-washed 5 times. The substrate p-nitrophenyl phosphate (pNPP) (Thermo Scientific, Waltham, MA, USA, Ref # 34045) was added with 1 mg/mL of DAE substrate buffer (ThermoFisher, Waltham, MA, USA, Cat # 34064) at 100 μL/well. After 15 min, the stop solution (1 M NaOH at 100 μL/well) was added to stop the reaction. The microplate ELISA reader (ELX-800, Tennessee, BioTek, Winooski, VT, USA) recorded OD values at 405 nm.

### 2.9. ELISA Sensitivity, Specificity, Positive Predictive Value (PPV), Negative Predictive Value (NPV), Likelihood Ratio Positive (LR+), Likelihood Ratio Negative (LR−) and Diagnostic Odds Ratio (DOR)

Sensitivity, specificity, positive predictive value (PPV), negative predictive value (NPV), likelihood ratio for a positive test result (LR+), likelihood ratio for a negative test result (LR−) and diagnostic odds ratio (DOR) were calculated according to the formulae in Table 1.

### 2.10. Statistical Analyses

All statistical analyses were performed using R-Studio version 1.0.143 (R Development Core Team, 2015). We conducted a generalised linear model (GLM) with a Gaussian error family with loglink function with the dependent variable being the percentage of ELISA-positive animals within the following categories (independent variables): species, sex and age. A separate GLM for each species, both with binomial error families and logit functions, was then used to test whether the percentage of ELISA-positive animals varied significantly in terms of age, sex, location of cyst and cyst viability. Chi-square tests determined the association of the disease with species, sex, location of cyst and age groups.

The Medcalc software (version 11.4.4.0) was used for the ROC curve line and interactive dot diagram. The ROC curve shows the sensitivity, specificity and criterion value (cut-off value) on the basis of which samples are declared positive or negative. The dot diagram displays (i) the separation of samples into positive (indicated as 1) and negative (indicated as 0) based on the gold standard (here, post-mortem examination); (ii) the cut-off value (indicated by a horizontal line) of ELISA separating ELISA-positive (above the line) and ELISA-negative (below the line) [36]. For comparing data in Medcalc software, we used “1” to indicate post-mortem positive samples (*n* = 38) and “0” to indicate post-mortem negative samples (*n* = 226). On the basis of two negative control samples (negative in post-mortem), we calculated the cut-off value to differentiate between sero-negative and sero-positive samples [37]. The mean OD value (0.136) of two negative controls was multiplied by 2.5 according to [38,39] to obtain the cut-off value of 0.340. In parallel, the OD values of all 264 samples were put into the Medcalc software while telling the software either positive and negative based on post-mortem examination. Based on this, the software also determined the cut-off value as being 0.341.

## 3. Results

Out of 264 bovines (128 cattle and 136 buffalo) examined at post-mortem, we found that 38 out of 264 (14.4%) contained hydatid cysts. All of these animals were subsequently confirmed as positive via serological examination using an ELISA kit based on the crude antigen (67 kDa) of BHCF. This ELISA approach also identified an additional 14 bovines, increasing the cystic echinococcosis infection rate to 52 out of 264 (19.6%).

### 3.1. Molecular Confirmation, Identification of Echinococcus-Specific Immunogen and BHCF ELISA

**Figure 1:**
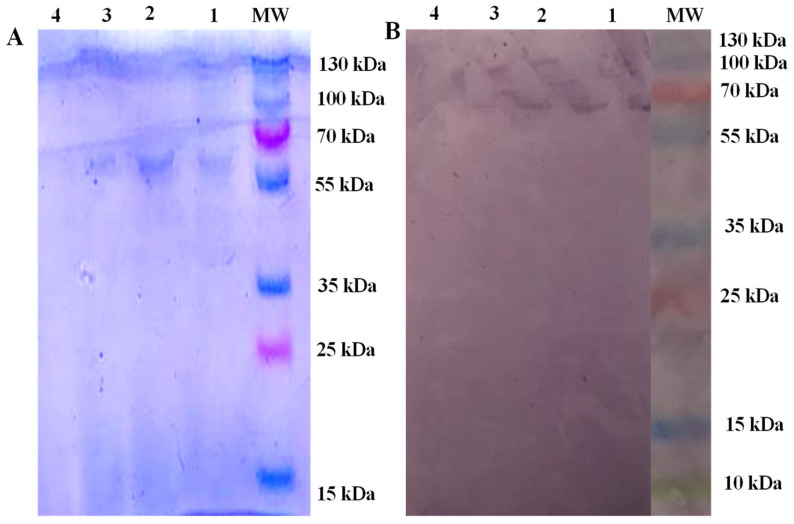
(**A**) Coomassie-blue-stained SDS-PAGE showing protein separation and identification of *Echinococcus granulosus* native antigen. Lane MW = protein marker, lanes 1–3 = samples, lane 4 = empty well. (**B**) Western blot of hydatid cyst fluids from slaughtered buffalo using sera from naturally infected buffalo and developed by an anti-bovine antibody conjugated with alkaline phosphatase. Lane MW = protein marker (run on the same gel as the test samples, but image cropped to remove samples unrelated to the current study), lanes 1, 2 and 3 = samples and lane 4 is an empty well.

For the antigen characterisation of BHCF, the hydatid cyst fluid collected from buffalo was confirmed to be *Echinococcus granulosus* based on species-specific primers targeting the mtDNA ND1 gene. The BHCF recovered from animals positive for echinococcosis, analysed via SDS-PAGE and stained with Coomassie blue, revealed an infected *E. granulosus* 67 kDa (IEg67 kDa) band and this was confirmed via WB analysis (Figure 1A,B). There was also a 130 kDa band present in the SDS-PAGE, potentially representing a dimer (Figure 1A) that was not explored further.

**Figure 2:**
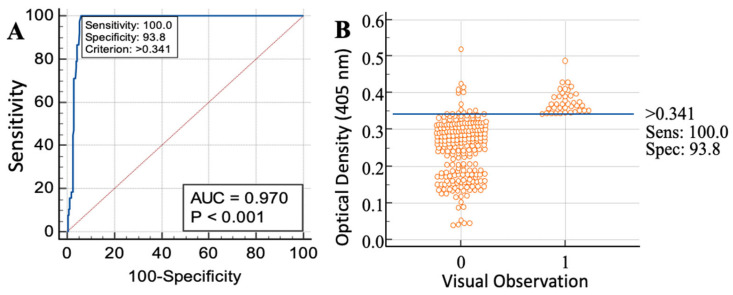
(**A**) Sensitivity, specificity and cut-off value of crude-antigen-based detection of *Echinococcus*-specific IgG antibodies in bovine sera. (**B**) Interactive dot plot for negative and positive sera.

The sensitivity and specificity of ELISA based on the crude antigen was 100% and 93.8%, respectively (Figure 2A,B).

For ELISA, the positive predictive value (PPV), negative predictive value (NPV), LR+, LR− and DOR are given in Table 2.

### 3.2. Echinococcus Occurrence

Based on ELISA, cattle had significantly higher infection levels than buffalo (GLM: std.err = 0.26, t = 2.5, *p* = 0.03). For both species, the number of infected individuals was positively associated with age (GLM: std.err = 0.09, t = 3.3, *p* = 0.009), and fewer males were infected than females (GLM: std.err = 0.25, t = −2.2, *p* = 0.05). The occurrence of cysts in cattle was significantly higher in the lungs (14.1%) compared to the liver (5.5%), whereas the reverse was true for buffalo (6.6% in liver and 2.9% in the lungs; χ^2^ = 10.69, *p* = 0.005). In each host species, neither location of cyst nor cyst viability were significantly related to host age nor gender (GLMs).

From post-mortem examination, the occurrence of cysts was higher in the lungs of cattle (72%) compared to the liver (28%), whereas the reverse was true for buffalo (30.7% in lungs and 69.2% in the livers; χ^2^ = 10.69, *p* = 0.005). Fertile cysts were more prevalent in lungs (64.5%) than in livers (28.6%), whereas sterile cysts were more frequently observed in the liver (71.4%) than in lungs (35.5%) (χ^2^ = 285.028 and *p* < 0.001). The percentage of fertile cysts was higher in buffalo (84.6%) than in cattle (80%), and there were significantly more sterile cysts in cattle (20%) than in buffalo (15.4%; χ^2^ = 285.028 and *p* = 0.06). The distribution of *Echinococcus*-positive animal sera is shown in Table 3.

## 4. Discussion

Cystic echinococcosis is a major disease in livestock and humans [40]. Its diagnosis in intermediate hosts is mainly based on necropsy procedures and in most cases the disease is asymptomatic in the early stages [41], so there is an urgency to develop alternative diagnostic methods [42], such as antibody detection through ELISA. Here, we identified and successfully deployed a potential candidate antigen, the iEg67 kDa crude antigen (from cystic fluid collected from buffalo liver) in a sero-diagnostic assay (Ab-detection ELISA) for the diagnosis of cystic echinococcosis in cattle and buffalo with much improved sensitivity and specificity. This antigen showed highly promising results as a candidate for ELISA kit development, and similar to previous studies (e.g., [43,44]) was more accurate than post-mortem examination, with very high sensitivity (100%) and specificity (93.8%) (Table 4). We also observed a 130 kDa band, possibly a dimer of iEg67 kDa, consistent with the previously reported dimerisation of *Echinococcus* antigens [45,46].

Previous studies have characterised the antigenic profile of hydatid cysts from cows, buffalo, sheep, goats and camels, and of humans infected with cystic echinococcosis, in different geographical areas to identify the potential diagnostic or vaccine candidate proteins [42,47,48]. The sensitivity and specificity of diagnostic tests used to include or exclude a disease (i.e., ‘rule in’ or ‘rule out’, respectively), for previous isolates of the HCF crude antigen, partially purified the crude antigen and recombinant antigens are compared in Table 5.

Factors influencing the sensitivity and specificity of ELISA include the source and quality of cyst fluid, the antigen purification process, laboratory techniques used for diagnosis and the target species/strains. The low sensitivity detected by Ibrahem et al. [49] might be related to the smaller size of their recombinant antigen potentially having fewer antigenic epitopes [49] compared to the native one. This notion is strengthened by Ibrahem et al. [49] who found that the sensitivity of recombinant-protein-based ELISA (25%) was lower compared to crude/native antigens from camel and sheep (see Table 5). Lower specificity might also arise due to the presence of other infections such as *Taenia hydatigena*, which can be mistaken as hydatid cysts, but would not have been detectable in an HCF-antigen-based ELISA [41]. Gatti et al. [37] used three forms of antigens (LHT, S2B and B) in their enzyme immunoassay (EIA) and recorded the sensitivity and specificity of each (see Table 4), concluding that either LHT or S2B should be used for the sero-diagnosis of cystic echinococcosis. Kandil et al. [50] used two types of antigens: protoscolex antigen and the germinal layer antigen of the hydatid cyst. They observed higher sensitivity (83%) and specificity (70.3%) in the protoscolex antigen than the germinal layer antigen [50] (Table 5). We used total hydatid cyst fluid for our crude HCF antigen preparation and obtained higher sensitivity and specificity values than in previous work; the reasons for this could be numerous.

The occurrence of hydatid cysts in Pakistan was determined in the current study to be 14.4% through post-mortem examination, and 19.7% when screened through the ELISA, similar to the 21% prevalence reported previously in cattle and buffalo from Punjab, India [51]. The increase in the detection rate of ELISA relative to the post-mortem between the current study and Yakubu et al. [43] can be explained by two complementary factors. Firstly, a known limitation of post-mortem lies in the lower detection probability for small developing cysts, possibly occurring more frequently when overall infection rate is higher in a population, as in [43]. Secondly, our ELISA approach (IgG Detection ELISA) did not allow us to differentiate between present and past infections, likely leading to the detection of recovered animals that would have remained undetected in PMs. ELISA can be further developed by targeting IgM for acute infections [52] and by the detection of the IgG titer in paired sampling [53] to have a clearer picture about active and past infections. The gap between the detection rate of ELISA and PM may increase when aged animals (with a higher expected proportion of recovered animals) are sacrificed and examined for the presence of cysts.

As previously reported by [54], infection prevalence was higher in female (18.75% post-mortem and 25% ELISA) than in male (9.2 and 13.3%) hosts. Pregnancy, parturition and lactation seem to enhance the risk of female infections [55]. Risk of infection also increased with age [56], presumably related to the increased exposure of older individuals. The current study also suggests that cattle are more prone to hydatidosis than buffalo (similar to [57], but in contrast to [54]). Apart from geographical location and temperature variation, the infection rate observed by [54] was influenced by the higher number of cattle amongst the slaughtered animals. In the current study, cattle had a higher occurrence of cysts in the lungs rather than the liver, with the reverse found in buffalo (as reported by [57,58,59], but in contrast to [60,61]. This difference may be due to host specificity, with one organ being more permissive to the development of cysts than the other, but this finding could potentially also be influenced by different strains of the parasite. With regard to hydatid cyst fertility and sterility, most studies on livestock populations in Punjab, Pakistan, have reported higher numbers of fertile than sterile cysts [29,47,58]. Variation in fertility may be due to differences in *Echinococcus* strain, time post-infection, development rate of the cysts and host species.

## 5. Conclusions

Cystic echinococcosis in food animals reduces their suitability for human consumption globally. Here, we show that an iEg67 kDa antigen identified from bovine hydatid cyst fluid is a suitable candidate for developing sero-diagnostic assays for cystic echinococcosis. An ELISA based on the use of the iEg67kDa antigen improved diagnosis accuracy for cystic echinococcosis in bovines compared to post-mortem examination. Our study showed 100% sensitivity and 93.8% specificity, an improvement compared with all previous studies. This approach can be used for large-scale sero-epidemiological studies of cystic echinococcosis in food animals (i.e., sheep, goat and camels) from diverse climatic conditions and agro-ecological zones, as well as for the screening of humans. We anticipate that this will provide increased information on the distribution and prevalence of cystic echinococcosis in animals and humans, informing on suitable control strategies and contributing towards the formulation of a One Health approach.

## Figures and Tables

**Table 1:** Definition and formulas to calculate sensitivity, specificity, PPV, NPV, LR+, LR− and DOR of ELISA, based on [35].

Name	Definition	Formula
Sensitivity	“The capability of a test to correctly identify the patients with disease”	a/(a + c) × 100
Specificity	“The potential of a screening test to identify the patients without the disease”	d/(b + d) × 100
Positive predictive value (PPV)	“Prediction of positive patients before test (identifying true positive)”	a/a + b
Negative predictive value (NPV)	“Prediction of negative samples before test (identifying true negative)”	d/c + d
Likelihood ratio positive (LR+)	“Ratio of probability that a positive test outcome would be expected in a patient with a disease to the probability that a positive test outcome would be expected in a patient without a disease”	(a/a + c)/(b/b + d)
Likelihood ratio negative (LR−)	“The ratio of probability of a patient testing negative who has a disease to the probability of a patient testing negative who does not have a disease”	(c/a + c)/(d/b + d)
Diagnostic odd ratio (DOR)	“Odds of a positive test in those with disease relative to the odds of a positive test in those without disease”	LR+/LR−

a = true positive, b = false positive, c = false negative and d = true negative.

**Table 2:** Sensitivity, specificity, PPV, NPV, LR+, LR− and DOR values of iEgELISA.

Sensitivity	Specificity	PPV	NPV	LR+	LR−	DOR
100%	93.8%	73.07	99.96	16.33	0.012	1360.83

**Table 3:** Distribution of *Echinococcus*-positive sera categorised by host species, sex and age group.

Age Class	Buffalo		Cattle	
	Female	Male	Female	Male
2–3 years	2/21 (9.5%)	0/23 (0%)	0/11 (0%)	4/28 (14.2%)
4–5 years	2/31 (6.5%)	3/19 (15.7%)	12/31 (38.7%)	4/22 (18.1%)
6–7 years	9/28 (32.1%)	1/4 (25%)	11/22 (50%)	4/14 (28.5%)

**Table 4:** Comparison of post-mortem and serological examination results among studies.

Host Species	Sample Size	Post-Mortem (%)	ELISA (%)	Antigen Used in ELISA	References
Cattle	128	19.5	27.3	Ammonium sulphate ((NH_4_)_2_SO_4_) precipitated HCF	Current study
Buffalo	136	9.5	12.5	Ammonium sulphate ((NH_4_)_2_SO_4_) precipitated HCF	Current study
Cattle	256	1.6	35.5	Freeze-dried precipitated HCF	[43]
Camel	304	14.14	52.6	Freeze-dried precipitated HCF	[43]
Camel	200	6	8	Phosphotungstic acid and 2M magnesium chloride used for HCF precipitation	[44]

HCF = hydatid cyst fluid.

**Table 5:** Comparison of sensitivity and specificity of iEgELISA based on crude antigen (this study) with results from previous work. Arrows denote the direction of change compared with values from the present study.

Sensitivity (%)	Specificity (%)	Host Origin	Antigen Size (kDa)	Type of Antigen	References
100	93.8	Buffalo	67	Crude antigen	Current study
82.8 ↓	62.5 ↓	Camel	Not mentioned	Crude antigen	[41]
79.3 ↓	75 ↓	Camel	Not mentioned	Partially purified crude antigen	[41]
36 ↓	93 ↓	Sheep	8–24	Crude antigen	[49]
25 ↓	99 ↑	Sheep	6	Recombinant antigen	[49]
96% ↓	71% ↓	Camel	Not mentioned	Crude antigen	[49]
99% ↓	90% ↓	Camel	Not mentioned	Camel antigen B	[49]
89.2 ↓	89.5 ↓	Sheep	8–12	Total hydatid liquid (LHT)	[37]
80.0 ↓	93.9 ↑	Sheep	16	Purified portion of total hydatid liquid (S2B)	[37]
86.4 ↓	92.8 ↓	Sheep	20–24	Purified antigen (B)	[37]
83 ↓	70.3 ↓	Camel	Not mentioned	Protoscolex antigen	[50]
46.5 ↓	41.7 ↓	Camel	Not mentioned	Germinal layer antigen	[50]

LHT = total hydatid liquid, S2B = purified portion of total hydatid liquid and B = purified antigen.

## Data Availability

The data will be available from the corresponding author upon request.
